# Insight into the Structure of Amyloid Fibrils from the Analysis of Globular Proteins

**DOI:** 10.1371/journal.pcbi.0020170

**Published:** 2006-12-15

**Authors:** Antonio Trovato, Fabrizio Chiti, Amos Maritan, Flavio Seno

**Affiliations:** 1 Consorzio Nazionale Interuniversitario per le Scienze Fisiche della Materia, Unità di Padova, Padua, Italy; 2 Dipartimento di Fisica “G. Galilei,” Università di Padova, Padua, Italy; 3 Dipartimento di Scienze Biochimiche, Università di Firenze, Florence, Italy; 4 Istituto Nazionale di Fisica Nucleare, Sezione di Padova, Padua, Italy; Harvard University, United States of America

## Abstract

The conversion from soluble states into cross-β fibrillar aggregates is a property shared by many different proteins and peptides and was hence conjectured to be a generic feature of polypeptide chains. Increasing evidence is now accumulating that such fibrillar assemblies are generally characterized by a parallel in-register alignment of β-strands contributed by distinct protein molecules. Here we assume a universal mechanism is responsible for β-structure formation and deduce sequence-specific interaction energies between pairs of protein fragments from a statistical analysis of the native folds of globular proteins. The derived fragment–fragment interaction was implemented within a novel algorithm, prediction of amyloid structure aggregation (PASTA), to investigate the role of sequence heterogeneity in driving specific aggregation into ordered self-propagating cross-β structures. The algorithm predicts that the parallel in-register arrangement of sequence portions that participate in the fibril cross-β core is favoured in most cases. However, the antiparallel arrangement is correctly discriminated when present in fibrils formed by short peptides. The predictions of the most aggregation-prone portions of initially unfolded polypeptide chains are also in excellent agreement with available experimental observations. These results corroborate the recent hypothesis that the amyloid structure is stabilised by the same physicochemical determinants as those operating in folded proteins. They also suggest that side chain–side chain interaction across neighbouring β-strands is a key determinant of amyloid fibril formation and of their self-propagating ability.

## Synopsis

In many fatal neurodegenerative diseases, including Alzheimer, Parkinson, and spongiform encephalopathies, proteins aggregate into specific fibrous structures to form insoluble plaques known as amyloid. The amyloid structure may also play a nonaberrant role in different organisms. Many globular proteins, folding to their biologically functional native structures in vivo, can be induced to aggregate into amyloid-like fibrils under suitable conditions in vitro. One hallmark of amyloid structure is a specific supramolecular architecture called cross-beta structure, held together by hydrogen bonds extending repeatedly along the fibril axis, but intermolecular interactions are yet unknown at the amino-acid level except for very few cases. In this study, the authors present an algorithm, called prediction of amyloid structure aggregation (PASTA), to computationally predict which portions of a given protein or peptide sequence forming amyloid fibrils are stabilizing the corresponding cross-beta structure and the specific intermolecular pattern of hydrogen-bonded amino acids. PASTA is based on the assumption that the same amino acid–specific interactions stabilizing hydrogen bond patterns in native structures of globular proteins are also employed by nature in amyloid structure. The successful comparison of the authors' prediction with available experimental data supports the existence of a unique framework to describe protein folding and aggregation.

## Introduction

An increasing number of human pathologies are associated with the conversion of peptides and proteins from their soluble functional forms into well-defined fibrillar aggregates [1,2]. The diseases can be broadly grouped into neurodegenerative conditions, in which fibrillar aggregation occurs in the brain, nonneuropathic localised amyloidoses, in which aggregation occurs in a single type of tissue other than the brain, and nonneuropathic systemic amyloidoses, in which aggregation occurs in multiple tissues [1,2]. The fibrillar deposits associated with human pathologies are generally described as amyloid fibrils when they accumulate extracellularly, whereas the term “intracellular inclusions” has been suggested to be more appropriate when fibrils morphologically and structurally related to extracellular amyloid form inside the cell [3].

Amyloid formation is not restricted, however, to those polypeptide chains that have recognised links to protein deposition diseases. Several other proteins that have no such link have been found to form fibrillar aggregates in vitro with morphological, structural, and tinctorial properties that allow them to be classified as amyloid-like fibrils [4,5]. This finding has led to the idea that the ability to form the amyloid structure is an inherent property of polypeptide chains, encoded in main backbone chain interactions. From a theoretical perspective it was also recently shown that simple considerations of geometry and symmetry are sufficient to explain, within the same sequence-independent framework, the emergence of a limited menu of native-like conformations for a single chain and of β-aggregate structures for multiple chains [6].

The generic ability to form the amyloid structure has apparently been exploited by living systems for specific purposes, as some organisms have been found to convert, during their normal physiological life cycle, one or more of their endogenous proteins into amyloid-like fibrils that have functional properties rather than deleterious effects [7–9]. Perhaps the most surprising of these functions is the ability of amyloid-like fibrillar aggregates to serve as a nonchromosomal genetic element. Proteins such as Ure2p and Sup35p *(Saccharomyces cerevisiae)* or HET-s *(P. anserina)* can adopt a fibrillar conformation that, in addition to giving rise to specific phenotypes, appears to be self-propagating, transmissible, and infectious [10].

In their soluble states, the proteins able to form fibrillar aggregates do not share any obvious sequence identity or structural homology to each other. In spite of these differences in the precursor proteins, morphological inspection reveals common properties in the resulting fibrils [11]. Images obtained with transmission electron microscopy or atomic force microscopy reveal that the fibrils usually consist of 2–6 protofilaments, each about 2–5 nm in diameter [12]. These protofilaments generally twist together to form fibrils that are typically 7–13 nm wide [11,12], or associate laterally to form long ribbons that are 2–5 nm high and up to 30 nm wide [13–15]. X-ray fibre diffraction data have shown that the protein or peptide molecules are arranged so that the polypeptide chain forms β-strands that run perpendicular to the long axis of the fibril [11].

Solid-state nuclear magnetic resonance (ss-NMR), X-ray micro- or nano-crystallography, and other techniques such as systematic protein engineering coupled with site-directed spin-labelling or fluorescence-labelling have transformed our ability to gain insight into the structures of fibrillar aggregates with residue-specific detail [16–29]. These advances have allowed us to go beyond the generic notions of the fibrillar appearance and presence of a cross-β structure. These studies have indeed allowed the identification of regions of the sequence that form and stabilise the cross-β core of the fibrils, as opposed to those stretches that are flexible and exposed to the solvent. In many cases, the arrangement of the various molecules in the fibrils has also been determined, clarifying the nature of the intermolecular contacts and the structural stacking of the molecules along the fibril axis. One frequent characteristic emerging from these studies, particularly for fibrils formed by long sequences, is the parallel in-register arrangements (PIRA) of β-strands in the fibril core [17–21,23–26,28], but antiparallel arrangements are also possible, especially for shorter strands [27,30].

At the same time, mutational studies of the amyloid aggregation kinetics revealed simple correlations between physico–chemical properties (charge, hydrophobicity, and β-sheet propensity) and aggregation propensities [31]. This allowed the development of different methods, which successfully predict aggregation-prone regions in the amino-acid sequence of a full-length protein [32–37]. All such approaches focus on predicting the intrinsic β-aggregation propensity of a sequence stretch using only the amino-acid sequence as an input. In [35] the possible parallel/antiparallel arrangement of the sequence stretch with itself was also taken into account. Molecular dynamics simulations of sequence fragments mounted on idealized β-strand templates, either parallel or antiparallel, were used to identify the most amyloidogenic fragments in a specific case [38]. A template amyloid structure based on PIRA is also employed in a very recent method for identifying fibril-forming segments [39]. A yet-unanswered question is why PIRA is found to be the most frequent arrangement of β-strands in the fibril core.

Here we introduce a computational approach by editing a pairwise energy function based on the propensities of two residues to be found within a β-sheet facing one another on neighbouring strands, as determined from a dataset of globular proteins of known native structures. We extract two different propensity sets depending on the orientation (parallel or antiparallel) of the neighbouring strands. Our method associates energy scores to specific β-pairings of two sequence stretches of the same length, and further assumes that distinct protein molecules involved in fibril formation will adopt the minimum-energy β-pairings in order to better stabilise the cross-β core.

A novel feature of our method is the ability to predict the registry of the intermolecular hydrogen bonds formed between amyloidogenic sequence stretches. In this way we can rationalise the observed tendency of proteins to assemble into parallel β-sheets in which the individual strands are in-register, contributing to form stackings of the same residue type along the fibril axis. Our algorithm is also able to correctly discriminate the orientation between intermolecular β-strands, either parallel or antiparallel. As a further demonstration of the robustness of the approach we will illustrate the ability of our algorithm to predict the portions of the sequence forming the cross-β core of the fibrils for a set of proteins, in excellent agreement with the experimentally determined amyloid structures, similar to previously proposed methods [32–37].

Our approach is based on the key assumption that a universal mechanism is responsible for β-sheet formation both in globular proteins and in fibrillar aggregates. The successful predictions obtained in this work suggest the validity of the above hypothesis in agreement with the unified framework presented previously [6].

## Results

### The Parallel In-Register Arrangement of β-Strands in the Amyloid-Like Fibrils

Based on the procedure described in detail in Materials and Methods and sketched in Figure 1, we can associate an energy score 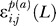 , from Equations 2 and 3, to the β-pairing of two sequence stretches chosen from distinct protein chains sharing an identical sequence. The pairing is specific since only pairs of residues facing each other in the corresponding register contribute to the energy score. All possible aggregation patterns are then defined in terms of the positions along the sequence *i,j,* the length *L,* and the relative orientation (either parallel or antiparallel) of the two sequence stretches participating in the pairing. We assume that the faithful repetition of this aggregating unit is at the basis of the assembly of polypeptide chains into amyloid fibrils, determining the highly regular cross-β core of the fibril.

**Figure 1:**
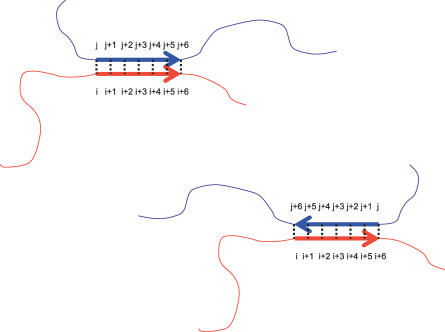
Sketch of the Method Presented in This Work Two identical protein chains are assumed to associate by means of an ordered pairing of two hydrogen-bonded β-strands of the same length (*L* = 7) while the remaining parts of the chains remain unstructured. All possible pairings can be obtained by sliding the two strand-forming regions (i.e., by varying *i* and *j*) along the corresponding sequences and by varying their length *L* and their relative orientations. The two possible orientations, parallel and antiparallel, for the same choice of sequence stretches participating in the pairing, are depicted. The corresponding pairing aggregation scores are obtained (Equations 2 and 3) by summing contributions for each of the *L* pairwise interactions between residues in front of each other in the paired strands, represented as dotted lines. Dotted lines do not represent hydrogen bonds. Interaction matrices (Equations 2 and Equation 1) are obtained from a statistical analysis of globular protein native structures, separately for parallel and antiparallel orientation. A term taking into account the entropy loss of the residues being ordered due to the pairing is further added.

We first analyse the properties of our energy function at the level of single pair energies 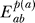 (see Equation 1). Residue pairs that appear from the analysis to possess low values of 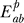 or 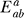 should then have a propensity to aggregate in the context of amyloid fibrils higher than other pairs. Figure 2 shows the distribution of the 210 entries for 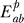 , 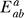 *,* and for the 20 in-register entries 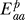 . All entries for both parallel and antiparallel pairing are shown in Table 1. Antiparallel pairing is favoured, on average, but the most favourable entries are found in the left tail of the parallel pairing distribution (with the only exception of the CYS–CYS antiparallel entry). Moreover, many of those are achieved for in-register pairings, notably for the hydrophobic residues VAL, ILE, and PHE. On the contrary, 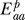 energies for charged and for some of the polar residues can assume significantly higher values. The highest 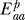 energy is obtained for PRO, as expected, since it breaks the regular pattern of main backbone hydrogen bonding.

**Figure 2:**
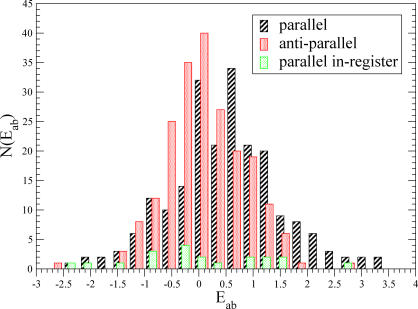
Histograms of the Energies for the Occurrence of Parallel and Antiparallel *β*-Pairing The third histogram shows the energies for the PIRA (a subset of the parallel case). The lowest energies correspond to the antiparallel arrangement of CYS–CYS and to the PIRA of VAL–VAL and ILE–ILE. Seventeen out of the 44 CYS–CYS residues found in native structures in anti-parallel β-pairing are forming disulfide bridges with each other, in agreement with previous reports [57,58]. Note that the energy for parallel arrangement of CYS–CYS is repulsive.

**Table 1:**
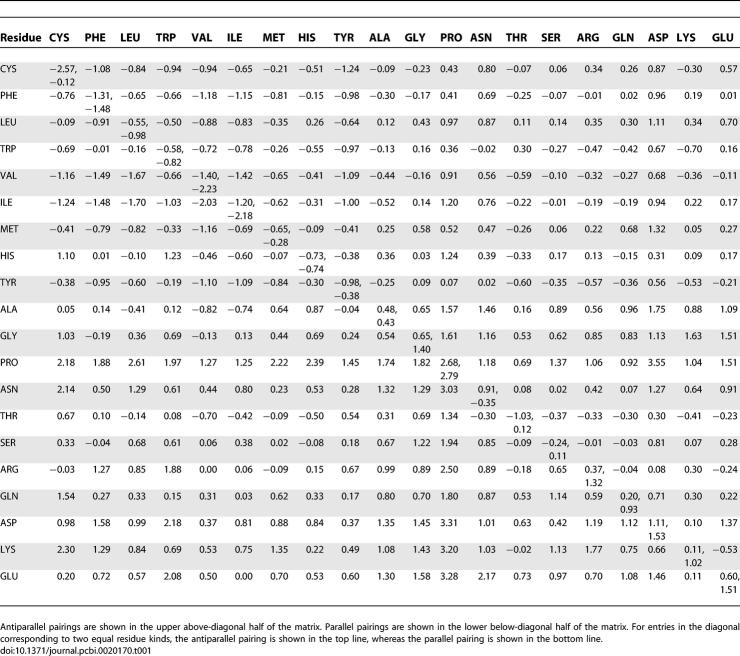
Entries for Both Parallel,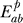 , and Anti-Parallel,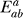 , Pairings, Computed as in Equation 1 (See Materials and Methods)

To verify whether the energies obtained with Equation 1 promote a general pattern in the aggregation, we use the sequence of the human amyloid β-peptide (Aβ_1–40_), a peptide known to be involved in Alzheimer disease and other pathological conditions such as hereditary cerebral hemmorhage with amyloidosis and inclusion-body myositis [2]. We are interested in rationalising on general grounds the competition between different registers in achieving the most favourable pairing. To average out as much as possible the influence of sequence specificity, we need to find a set of different minimum energy pairings. For fixed *L* and |*i* – *j*|, we slide the β-pairing segments along the sequence looking for the minimum energy pairing in both the parallel and antiparallel orientations (for the analysis shown in Figure 3 we consider the length independent energy term 
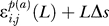
 ). The minimum energies collected in this way are then averaged over different segment lengths (4 ≤ *L* ≤ 23) for a fixed value of |*i – j*|, yielding a mean value that is plotted as a function of |*i – j*| in Figure 3. As a matter of fact, the in-register parallel alignment (|*i – j*| = 0) is considerably more favourable than any other out-of-register parallel alignment (|*i – j*| ≠ 0). We interpret oscillations in the curve for parallel pairings as a signature of some degree of pattern repetition in the sequence. On the other hand, (|*i – j*| = 0) is the preferred pairing also for antiparallel orientation, but in this case the average minimum energy exhibits a linear increase with |*i – j*|. All these features are consistently retrieved in all sequences analysed in this work (unpublished data), whereas the existence and the values of the “gap” between the |*i – j*| = 0 parallel and antiparallel depends crucially on the specific sequence (see Table 2).

**Figure 3:**
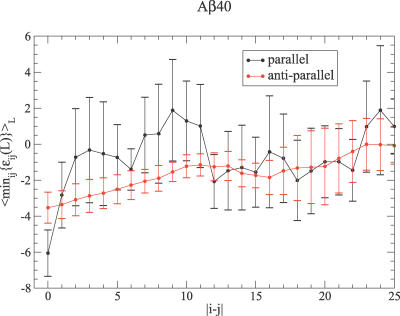
Plot of the Average over *L* of min*_ij_* ɛ*_ij_* (*L*) as a Function of |i − j|, Obtained with the *A*β40 Peptide for Both Parallel and Antiparallel Orientation Bars represent the standard deviations of the minimum energies obtained for different segment lengths *L*. The linear increase with |*i* − *j*| of the antiparallel curve can be explained in the following way. If |*i* − *j*| = *l*, with *l ≤* L, [(*L* − *l* ) / 2] terms are repeated twice in the last sum of the right hand side of Equation 2 ([*x*] is the integer part of *x*) so that the number of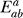 values to be searched for low values is [(*L* + *l* + 1)/2]. Since the smaller this number the easier to find a good pairing, antiparallel pairing is more and more favoured as *l* ≤ *L* is more and more decreased until for *l* = 0 one gets the most favourable antiparallel pairing.

**Table 2:**
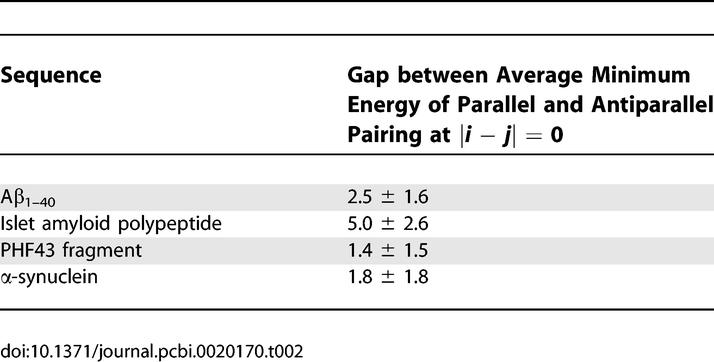
Energy Difference between Average Parallel and Antiparallel In-Register (|*i* − *j*| = 0) Pairings (See Figure 3)

Our results show that on average the assembly of Aβ_1–40_ molecules with PIRA of sequence segments is favoured over both antiparallel and parallel out-of-register arrangements. ss-NMR and site-directed spin labelling experiments indeed show that amyloid fibrils from Aβ contain such a parallel in-register stacking of β-strands contributed by distinct molecules [17,18]. Similar results are obtained when computing the sequences of amylin, α-synuclein, and the PHF43 segment of tau protein (unpublished data), again in agreement with the experimental results [19–21,23]. For the Aβ_1–40_ peptide and for the islet amyloid polypeptide, PIRA is clearly preferred over the antiparallel one within this analysis (Table 2). On the other hand, the preference is milder for the PHF43 fragment of the tau protein, and for human α-synuclein, being within the standard deviation of the energies employed for the average, as shown in Table 2.

The behaviour of the two curves shown in Figure 3 can be understood on the basis of simple statistical considerations. The problem consists in finding several low-energy pairings in a row. For a generic out-of-register parallel arrangement, the lowest 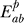 values need to be found within all 210 possible entries. Therefore, the probability of finding several consecutive low-energy pairings is indeed quite low, independently of the sequence distance |*i – j*| between the segments (as long as |*i – j*| ≠ 0). On the other hand, the search problem is much easier in the case of in-register parallel pairing (|*i – j*| = 0), since the lowest pairing energies need to be found only within the 20 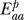 entries (see Figure 2). Therefore PIRA is favoured, with respect to other parallel alignments, because many of the most favourable entries can be found more easily.

In the case of antiparallel arrangement, the search always has to be performed among 210 entries, but a symmetry effect favours the |*i – j*| = 0 register. Indeed, when two overlapping sequence segments are aligned in antiparallel manner, some pairings are repeated twice (see the antiparallel case in Figure 1 with *j* = *i* ). The number of low-energy pairings to be found is thus effectively reduced. The extent of this reduction is proportional to the length of the overlapping portion, thus explaining the linear increase with |*i – j*| of the antiparallel curve in Figure 3. (Further details can be found in the Figure 3 legend.)

We remark that the above general arguments rely on the fact that the most favourable entries do indeed correspond to PIRAs, due to the stacking of hydrophobic and hydrophilic residues. In other words, PIRA provides a natural way of maximizing the number of favourable stacking interactions, lining up hydrophobic and hydrophilic residues in long rows along the fibril axis. Any other out-of-register parallel arrangement will most likely disrupt such an ordered pattern of stabilizing interactions.

### Prediction of Alignment Orientation for Fibril-Forming Peptides

We employ prediction of amyloid structure aggregation (PASTA) to predict the orientation between β-strands in fibrillar structures formed by short, previously investigated peptides. In all cases we assume the full peptide length is involved in the β-core of the fibril, so that we simply compare the energy score of the parallel and antiparallel β-pairings of the full segment with itself. Results are shown in Table 3, showing in the three considered cases that PASTA correctly identifies the experimentally determined orientation as the minimum energy pairing. To our knowledge, the first two peptides are the only cases of a detailed atomic resolution achieved for a fibrillar structure obtained by means of X-ray diffraction from microcrystals. GNNQQNY is a fragment from the yeast prion protein Sup35 displaying a parallel orientation between β-strands within the same β-sheet [28]. KFFEAAAKKFFE is a peptide explicitly designed to form amyloid-like fibrils and was shown to be composed of antiparallel β-sheets [27]. KLVFFAE is the (16–22) fragment of the human Aβ_1–40_ amyloid peptide, whose β-sheet structure was indicated to be antiparallel by ss-NMR data [40]. In the latter case it is remarkable that PASTA recognises the tendency of the short (16–22) fragment to form antiparallel β-sheets while at the same time predicting the correct in-register parallel alignment for the full sequence (see below).

**Table 3:**
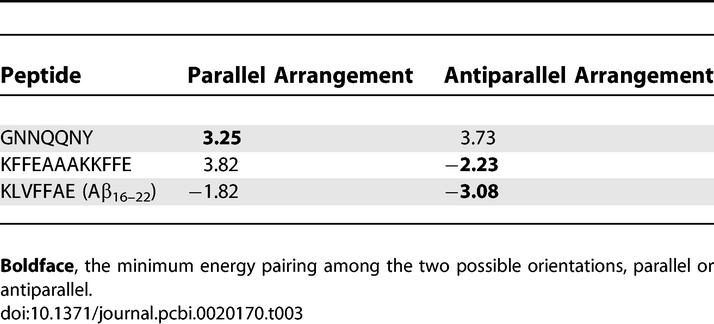
Pairing Energies Predicted by Equations 2 and 3 for the Listed Peptides, Assuming the Full Peptide Length Is Involved in a β-Pairing with Itself

### Prediction of Specific Pairings and Sequence-Aggregation Propensities

We employ PASTA to identify the regions of the sequence-promoting aggregation for five natively unfolded systems. These include human Aβ_1–40_, human α-synuclein, the human islet amyloid polypeptide, the PHF43 fragment from human tau, and the HET-s prion domain protein from P. anserina. We decided to perform the analysis on such systems rather than on globular proteins because our analysis utilises values of intrinsic propensity to aggregate residue pairs and does not take into account the presence and type of secondary and tertiary structure in the analysed polypeptide chain. Indeed, it is well-known that the presence of structure in the initial nonaggregated state of the protein is an important determinant of aggregation and reduces dramatically the aggregation propensity of the structured regions [41]. In addition, the five natively unfolded systems analysed here were chosen because their aggregation-promoting regions were also determined experimentally, allowing our predictions to be directly tested.

The energy functions introduced in Equations 2 and 3 can be used to compare different segment lengths, and we will first list the three pairings yielding the minimum energy when looking among all possible segment lengths. (By definition the energy of a nonaggregating system is zero.) The results are summarized in Table 4. We then use the single-residue propensity *h*(*k*) defined in Equation 5 to take into account other low-energy pairings that could be close competitors of the lowest-energy pairing.

**Table 4:**
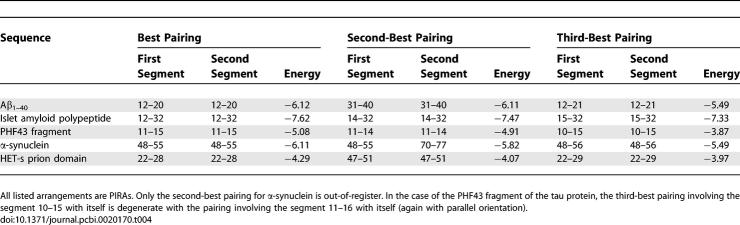
Best Pairing Energies Predicted by Equations 2 and 3

#### Human amyloid β-peptide.

We first apply PASTA to study Aβ_1–40_. It is known by proline-scanning mutagenesis and quantitation of fibrils by Congo red binding [42], ThT binding, electron microscopy, and SDS-Page [43], ss-NMR (17) and site-directed spin labelling [18] that the regions of the sequence involved in β-aggregation are approximately the segments 12–24 and 30–40 (the boundaries of the two regions vary somewhat in the various reports). Both segments are almost exactly predicted and are found as minima closely competing with each other. In Figure 4A we are plotting *h*(*k*) for Aβ_1–40_. We see that in the region 12–20 and 31–40 the propensity is very strong, in almost perfect agreement with the experimental prediction, whereas it is negligible in the other parts of the protein. In both cases PIRA is predicted in perfect agreement with experimental data [17].

**Figure 4:**
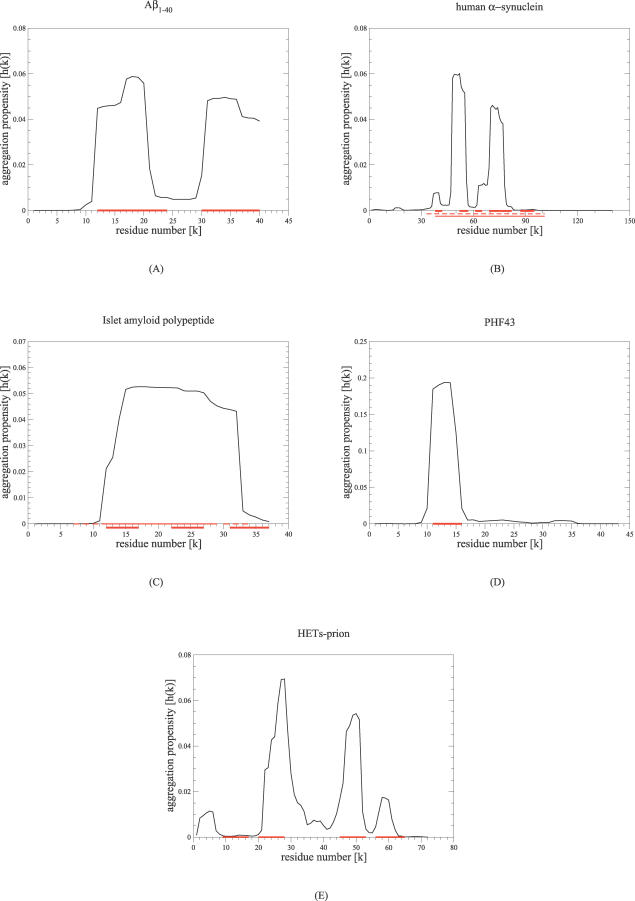
Amyloid Propensity Plots for the Proteins Studied in This Work (A) Plot of amyloid propensity *h*(*k*) (Equation 5) for the human amyloid β**-**peptide. The sequence regions involved in β-strands according to ss-NMR experiments [17] are represented by a thick red line along the *k-*axis. (B) Same as in (A) but for the protein human *α*-synuclein. Thick red bars mark sequence stretches involved in β-strands according to ss-NMR experiments [23]. The thin red bars show the whole sequence portion found to be in PIRA, according to site-directed spin-labelling, solid line [19], and found to participate in main backbone hydrogen bonding according to hydrogen–deuterium exchange, dashed line [22]. The two experimentally determined portions differ only in the location of the initial boundary. (C) Same as in (A) but for the subsection islet amyloid polypeptide. The thin red line shows the whole sequence portion found to be in PIRA according to site-directed spin-labelling experiments, with the dashed portions representing the uncertainty on boundary location [20]. Thick red bars show the sequence portions proposed to participate in β-strands according to a structural model based on a serpentine PIRA [24]. (D) Same as in (A) but for the PHF43 fragment from the fetal form of human tau. The thick red line shows a local sequence motif identified to be crucial for β-aggregation [46]. (E) Same as in (A) but for the HET-s prion domain protein from *P. Anserina*. The red bars show sequence portions involved in β-strands as determined by fluorescence studies, quenched hydrogen exchange NMR, and ss-NMR (29).

#### Human α-synuclein.

This protein is involved in Parkinson disease and in dementia with Lewy Bodies [2]. By synthesising peptides of various lengths and quantifying their aggregation using HPLC and circular dichroism, the region 63–78 has been proposed to be involved in aggregation [44,45]. More recent experimental studies employing ss-NMR have allowed the identification of several sequence portions involved in β-strand formation within the fibrils [23]. These are shown as thick red bars in Figure 4B, together with the aggregation profile predicted by our algorithm. Four out of five of the experimentally determined sequence stretches are correctly identified by PASTA. The overall arrangement is parallel in-register, as determined by site-directed spin-labelling studies [19]. PASTA correctly finds the best minimum for a parallel in-register pairing, but the second-best pairing is a parallel out-of-register one. Looking at the segments involved, which are VVHGVATV (48–55) and VVTGVTAV (70–77), we realize that this is due to a strong pattern repetition. Five out of eight residues are matched for an in-register alignment, including the four valines that are most responsible for the low pairing energy. In Figure 5 we show the β-pairing contact map *h*_2_(*k*,*m*), where a compendium of the general features predicted by PASTA can be found. The strongest signal is for PIRA, but parallel out-of-register arrangement is also selected in the presence of repetition of sequence patterns along the chain. Weak signals are also present for antiparallel arrangement, which would take place between identical sequence stretches, as predicted on general grounds.

**Figure 5:**
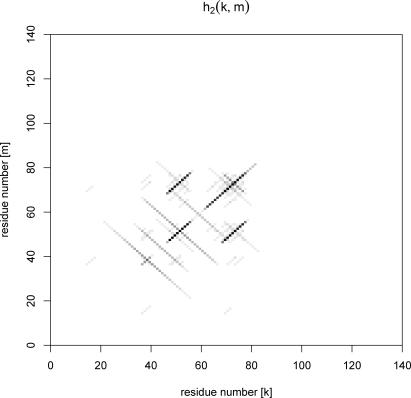
β-Pairing Contact Map (Equation 6) for Human *α*-Synuclein This picture was obtained with *λ* = 1.5, for a better visualization of the competition between the best pairings.

#### Islet amyloid polypeptide.

The 37-residue islet amyloid polypeptide is the major component of pancreatic amyloid deposits, which are the hallmark of noninsulin—dependent (type II) diabetes mellitus. We plot *h*(*k*) in Figure 4C. Again there is quite a good agreement with site-directed spin-label experiments (20), which show parallel in-register aggregation in the region 12–29. It should be remarked that in this case, unlike for Aβ_1–40_, PASTA clearly signals the existence of a single continuous pairing. In a recently proposed model, resulting from a number of experimental constraints, residues 12–17, 22–27, and 31–37 are proposed to form β-strands in a serpentine arrangement in each molecule, with very short loops connecting them [24]. This structural arrangement is repeated for each peptide molecule along the fibril axis so that the parallel in-register orientation is maintained [24]. The short length of the loop may make it difficult to distinguish between a single continuous pairing and three very-nearby short pairings.

#### PHF43 fragment from the fetal form of human tau.

Filamentous inclusions from tau proteins are present in numerous neurodegenerative diseases, including Alzheimer disease and frontotemporal dementia with Parkinsonism linked to Chromosome 17 [2]. The region, found experimentally to be involved in aggregation within the tau fragment PHF43, is the segment 11–16, as identified by means of spot membrane–binding assay [46]. A good agreement is again found between these experimental data and those found with our prediction, as shown by both the minimum energy pairings listed in Table 4 and the plot of *h*(*k*) in Figure 4D. The arrangement is also correctly predicted to be parallel in-register, as determined by site-directed spin-labelling coupled with EPR methods [21].

#### HET-s prion domain fragment from P. anserina.

The prion form of the protein HET-s is involved in a programmed cell death mechanism called heterokaryon incompatibility [47,48]. The recombinant HET-s prion domain (fragment 218–289) can form amyloid-like fibrils in vitro and induce prion phenotypes in a host cell [49]. Recent experiments employing fluorescence studies, quenched hydrogen exchange NMR, and ss-NMR [29] determined four sequence portions involved in β-strand structure within the fibrils, shown as red bars in Figure 4E, together with the aggregation profile predicted by our algorithm. PASTA correctly predicts four sequence stretches to be involved in β-aggregation, placing three of them in good agreement with experiments. The peculiar arrangement suggested by Ritter et al. on the basis of their experimental data is parallel but not in-register, pairing different portions of the same chain [29]. The method described in this work is based on the assumption of interchain pairing. Further studies are being carried out to extend our algorithm to intrachain pairing as well.

## Discussion

We introduced a pairwise energy function based on the propensities of two residues to be found within a β-sheet facing one another on neighbouring strands, as determined from a dataset of globular proteins of known native structures. Such energy function was incorporated within an algorithm able to predict amyloidogenic sequence stretches, as well as the registry of the intermolecular hydrogen bonds formed between them. The latter type of prediction is a novel feature of our approach.

For a set of natively unfolded proteins involved in the formation of amyloid fibrils, we correctly predict their observed tendency to assemble into parallel β-sheets in which the individual strands are in-register. Our algorithm is also able to correctly determine the orientation between β-strands in the fibrils, either parallel or antiparallel, as shown by a comparison with fibrillar structures formed by short peptides determined experimentally at the atomic level.

Our energy function predicts that PIRA is favoured on general grounds, with respect to other parallel out-of-register alignments, because the most favourable β-pairing found in globular proteins is indeed parallel and obtained for hydrophobic pairs sharing the same residue kind. Even though such parallel in-register pairing can be unfavourable for other residues (especially charged ones), PIRA by itself constrains the search for good pairs in a much smaller set than for out-of-register arrangement. A similar, yet milder, effect induced by pairing statistics is detected for antiparallel arrangement, favouring the case in which the latter is achieved between *identical* sequence stretches. Parallel arrangement is generally favoured over antiparallel, but in some cases sequence specificity can override this tendency, as in the case of short peptides. Out-of-register parallel arrangement is also predicted as a good competitor in the presence of repeated (periodic) patterns in the sequence, which actually occur in several prion proteins, both in mammals and in fungi.

Our algorithm was also used to predict the portions of the sequence, for an initially unstructured polypeptide chain, that form the cross-β core of the fibrils. A good agreement with the experimental information available on amyloid structures, similar to other proposed methods [32–37], was found for human Aβ_1–40_, α-synuclein, islet amyloid polypeptide, a fragment from human tau, and the prion domain of HET-s from P. anserina.

The results obtained in this work, besides rationalising on general grounds the common occurrence of PIRA in amyloid fibrillar structures, suggest two important conclusions. First, the existence of a preferred β-pairing is an important determinant of the self-propagating nature of amyloid fibrils and of the difficulty of these to seed the fibrillar state in proteins that have even subtle differences in sequence, a phenomenon associated with the species barrier in prion transmissibility. Moreover, the polymorphism often observed for amyloid fibrils [15,50], leading to the existence of different prion strains [10], might be explained by the competition between different low-energy β-pairings that are realizable for the same sequence.

The notion of a preferred β-pairing is the simplest one that can be put forward to account for the self-complementation of protein molecules on a structural basis [51]. It can be seen as a way of reconciling the roles of side chains in driving specific aggregation and of main backbone interactions in determining the general tendency of polypeptide chains for fibril formation. The knowledge-based energy function introduced in this work describes how side chain–side chain interactions between residues facing each other modulate the main chain hydrogen bond energy common to all residues. Stacking of hydrophobic residues [27] or hydrogen bonding between side chain groups [28] will favour PIRA, whereas electrostatic repulsion between charges of the same type disfavours it. All such interactions are captured within our knowledge-based approach. A determinant of self-complementation that we neglect in our simple scheme is the steric interdigitation between different sheets forming the fibril core [39]. However, the good performance of our algorithm shows that sequence information is already relevant at the level of β-strand pairing within the same sheet.

As a second important conclusion, the fact that the whole computational approach is derived from the knowledge of globular proteins underscores the universality of the physico–chemical mechanisms underlying amyloid fibril formation. Moreover, it indicates that the structure and stabilising interactions existing in the apparently monotonous amyloid or amyloid-like fibrils are of the same essential nature as those determining structural and functional diversity in globular proteins.

## Materials and Methods

### Knowledge-based pair potential.

We derive an energy function for specific β-aggregation using the top500H database [52]. It is a nonredundant specially refined set of 500 high resolution X-ray crystallographic structures of globular proteins, where hydrogen atoms were also reconstructed. These proteins include all-α, all-β, α/β, and α + β proteins, and their structures are deposited in the Protein Data Bank. All occurring instances, *n_ab_,* of a given *ab* residue pair are partitioned (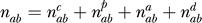 ) into four different classes according to whether the two residues are facing each other on neighbouring parallel β-strands (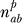 ) or on neighbouring antiparallel β-strands (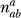 ), and whether the distance between their *C^α^* atoms is less than 6.5 Å—without participating in a ordered β-geometry (generic bulk contacts 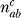 )—or more than 6.5 Å (noncontacting disordered pairs 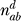 ). All pairs are included in the count, except those formed by consecutive residues along the protein chain. The participation to either parallel or antiparallel β-bridges is assessed by using the DSSP algorithm [53], but with a slightly stricter electrostatic energy threshold of −1 Kcal/mol to assign hydrogen bonds. (The distribution of such energies obtained from the Richardson set peaks around the value of −2.4 Kcal/mol, but increases again for values higher than −1 Kcal/mol, unpublished data).

Energies can be assigned to the occurrence of parallel β-pairing and antiparallel β-pairing for two amino acids of type *a* and type *b,* by assuming that the database of protein native structures is a system in thermodynamic equilibrium at a single temperature, assumed to be roughly constant for all the proteins in the database [54]. Upon further assumption that correlations between different pairings can be neglected within single proteins in the database [55], the *propensity, p_ab_(x),* of the *ab* pair to be found in one of the four pairing types, *x,* is given by the Boltzmann factor, *p_ab_*(*x*) *= exp*(−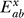 ). The *E* 's are energy differences, measured in units of thermal energy, between the native and the reference state with respect to which propensities are computed [54].

Propensities are defined as the ratio of the observed frequency over the expected probability in the reference state, which is in turn estimated as the frequency observed over all pairs.

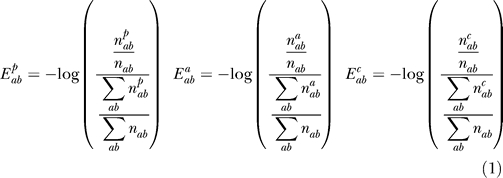



A similar expression yields the energy 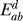 *,* which should be assigned to a noncontacting pair *ab*. Since the numbers 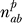 *,* 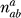 *,* and 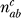 can be very small (or even zero in some special cases involving PRO and CYS), we used an averaging procedure to decrease statistical error [33]. Hence, for example, 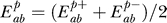 , where 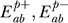 , are the energies obtained from Equation 1 when adding (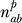 → 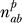 + 1, 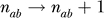 ) or subtracting (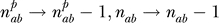 ), a single event, to the observed number of cases (whenever 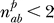 , 0.5 is used in place of 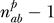 ). Statistical potentials describing residue pair correlations within β-sheets were developed in the context of structure prediction, limiting the total ensemble of residue pairs to those in which both residues participate in a β-structure [56–59]. Our derivation instead places all residue pairs in the total ensemble.

### β-pairing energy function.

Our aim is to predict the specific aggregation pattern of a pair of identical proteins of *N* amino acids {*a_k_*}_1≤*k*≤*N*_ *,* as determined by the specific β-pairing (either parallel or antiparallel) of the sequence stretch of length *L,* beginning at position *i* on the first chain, with the sequence stretch of the same length, beginning at position *j* on the second chain. We assume throughout the rest of this work that only a single stretch per sequence participates in the β-pairing and that all other residues (from 1 to *i* − 1 and from *i* + *L* to *N* for the first chain and from 1 to *j* − 1 and from *j* + *L* to *N* for the second chain) are not involved in aggregation and are found in a disordered noncompact conformation. We assume further that the energies 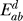 of all pairs involving these latter residues can be neglected, since 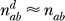 and 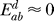 . Remaining pairs whose residues are both present in the β-aggregating stretches but not specifically paired with each other are assumed to be noncontacting as well. We verified that the results we present in this work do not change upon inclusion of noncontacting pair terms. The overall pairing aggregation energy for a given parallel/antiparallel pattern is then determined only by residue pairs mutually involved in the ordered β-pairing, and can be written, by assuming they do so independently of one another, as

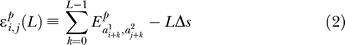


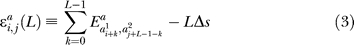
where the overscripts 1 and 2 correspond to the first and second chain, respectively, and Δ*S* = *L*Δ*s* is the entropy loss due to the β-ordering of the *L* residue pairs, with Δ*s* corresponding to the average entropy loss per residue pair. Due to the many approximations involved in the standard derivation of statistical potentials, the latter extensive term might actually compensate for any bias introduced with the choice of the reference state, making its a priori evaluation too difficult. Therefore we set Δ*s* = −0.2 throughout all our work on a purely empirical basis. The proper introduction of sequence specific 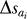 might certainly improve the quantitative agreement with experimental observations, but we chose to keep our energy-scoring function as simple as possible to directly test the relevance of β-pairing specificity in dictating aggregation patterns. Since the computation of energy scores 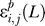 and 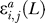 involves a summation over only *L* terms, it can be easily performed on a genome-wide scale.

### Sequence-dependent aggregation propensities and contact maps.

To take into account in a more complete manner all possible pairing energies close to the minimum, we introduce an “ordered β-pairing partition function”:

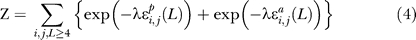
where we set λ = 2.0 as an adimensional factor setting the energy scale. Parameters Δ*s* and *λ* need not to be fine-tuned and can be changed within a 20% range without affecting the final results. The partition function (Equation 4) allows a better one-dimensional visualization of the results by defining a position-based “amyloid propensity”

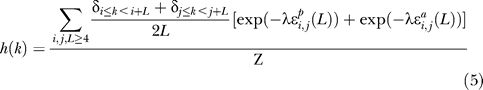
where δ*_i ≤ k_*_ < *i* + *L*_ = 1 if residue *k* belongs to the *L*-stretch going from *i* to *i* + *L*−1 and δ*_i ≤ k_*_ < *i* + *L*_ = 0 otherwise. Note that *h*(*k*) is a probability since 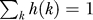 . It tells how a given residue is more likely to aggregate in an ordered β-structure with respect to others.

A more complete piece of information that can be extracted from the method is the normalized two-dimensional probability *h*_2_(*k*,*m*) of two given residues found paired to each other within an ordered β-structure. It is given by

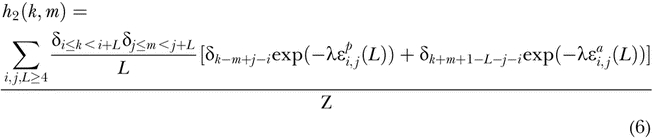
 where *k* and *m* label residues in two different chains and *δ_k_*_− *m* + *j* − *i*_ = 1 if *k* – *m* + *j* − *i* = 0, and 0 otherwise. Based on *h*_2_(*k*,*m*), a β-pairing contact map can be produced where the orientation (parallel or antiparallel to the diagonal) and the register of the best pairings is easily traced out (see Figure 5).

We name the full procedure described in this section PASTA.

## Abbreviations

PASTA: prediction of amyloid structure aggregation
ss-NMR: solid-state nuclear magnetic resonance
PIRA: parallel in-register arrangement
